# Supplementary material for: Homology-Directed Repair of a Defective Glabrous Gene in Arabidopsis With Cas9-Based Gene Targeting
Source: Front Plant Sci. 2018 Apr 5;9:424. doi: 10.3389/fpls.2018.00424 (PMC5895730; doi:10.3389/fpls.2018.00424)
Supplement: Supplementary file 1 [file Data_Sheet_1.pdf]

Supplementary Material

Homology-Directed Repair of a Defective *Glabrous* Gene in *Arabidopsis* With Cas9-Based Gene Targeting

Florian Hahn[1], Marion Eisenhut[1], Otho Mantegazza[1], Andreas P.M. Weber[1]*

[1]Institute of Plant Biochemistry, Cluster of Excellence on Plant Science (CEPLAS), Center for Synthetic Life Sciences (CSL), Heinrich Heine University, Düsseldorf, Germany

*** Correspondence:** Andreas P.M. Weber, Andreas.Weber@uni-duesseldorf.de

1 Supplementary Figures and Tables

1.1 Supplementary Figures

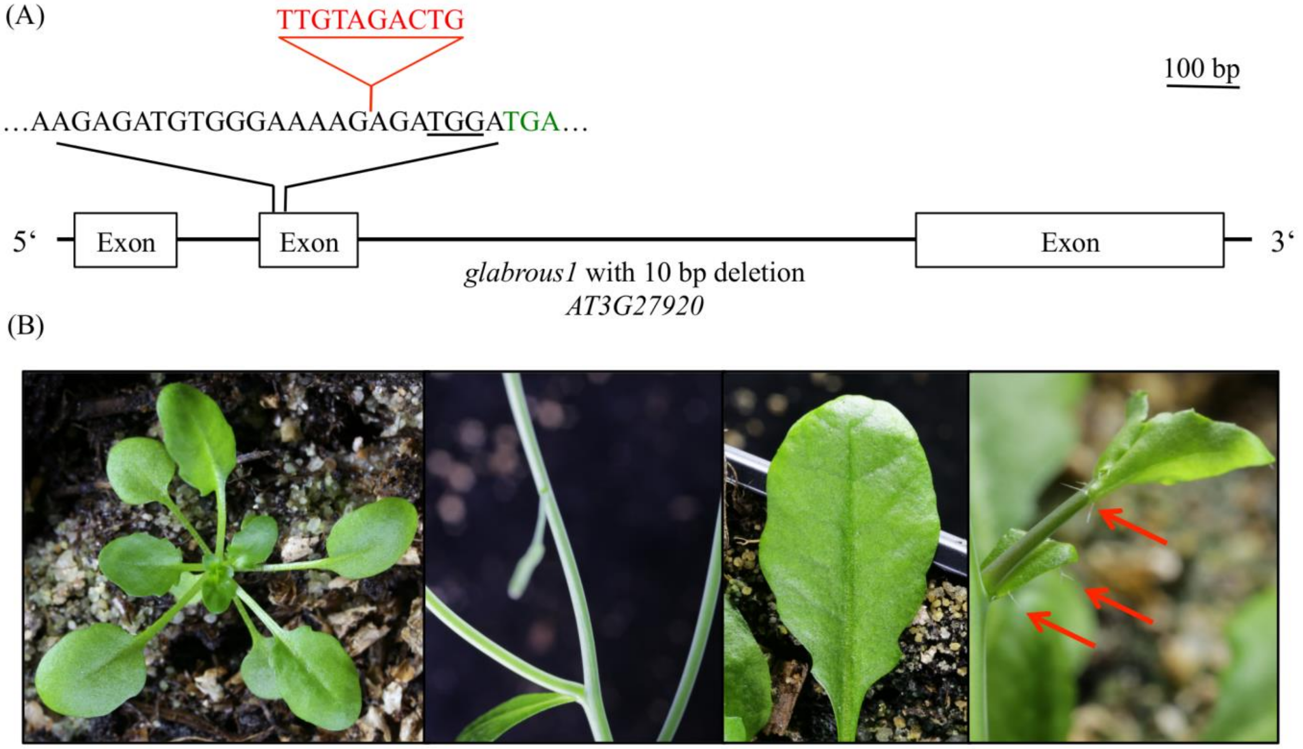

Supplementary Figure S1: A trichomeless *Arabidopsis gl1* mutant is a suitable model for Cas9-mediated repair. In previous experiments, we generated an *Arabidopsis* line with a 10 bp deletion (red) in the second exon of the *GL1* gene (A). The deletion is located three nucleotides in front of a NGG motive (underlined), which functions as PAM motive in *S. pyogenes* Cas9 systems. The deletion leads to a premature STOP codon (green) that disrupts the protein function. Plants carrying

the mutations show no trichomes on the leaf surface of rosette leaves and on stems. (B) Some *gl1* mutant plants show single trichomes (red arrows) on leaf margins and on the transition to the petiole, but only on leaves that derive from the stem.

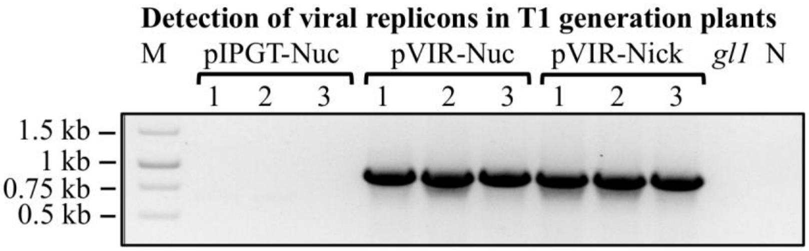

Supplementary Figure S2: Detection of viral replicons in T1 generation plants transformed with pVIR-constructs. We analyzed replicon formation on leaf gDNA of three independent T1 *Arabidopsis* lines transformed with pIPGT-Nuc, pVIR-Nuc, and pVIR-Nick, respectively, using a primer pair that only yields a PCR amplicon on circular replicons. DNA of a *gl1* plant was used as negative control. Replicons could be detected in all T1 plants transformed with a pVIR-construct at the expected size of 919 bp. Image of agarose gel was color inverted for better visibility. M = Marker, N = water control.

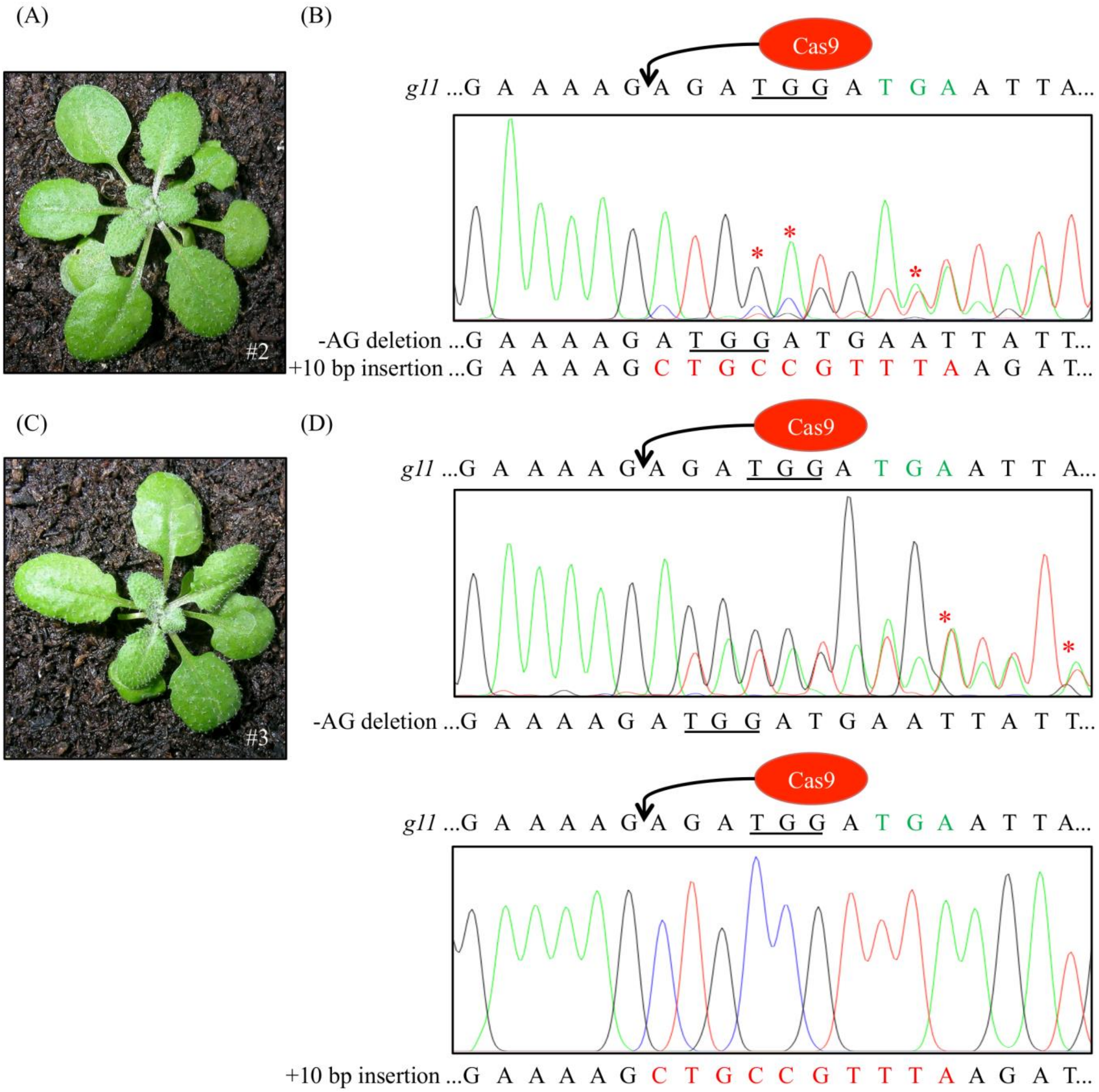

Supplementary Figure S3: Sequence analysis of T3 trichome plants #2 and #3. (A) Image of trichome-bearing plant #2. (B) Sequence analysis of *gl1* gene of plant #2. Multiple peaks that indicate chimerism and unfixed mutations are marked with red asterisks. (C) Image of trichome-bearing plant #3. (D) Top: Sequence analysis of *gl1* gene of plant #3. Bottom: Sequence of a subcloned *gl1* gene of plant #3. Sequence of the dysfunctional *gl1* gene is given on top of each sequencing histogram for comparison. Peak colors: Adenine = green, cytosine = blue, guanine = black, thymine = red; PAM sequence underlined, premature STOP codon marked in green letters.

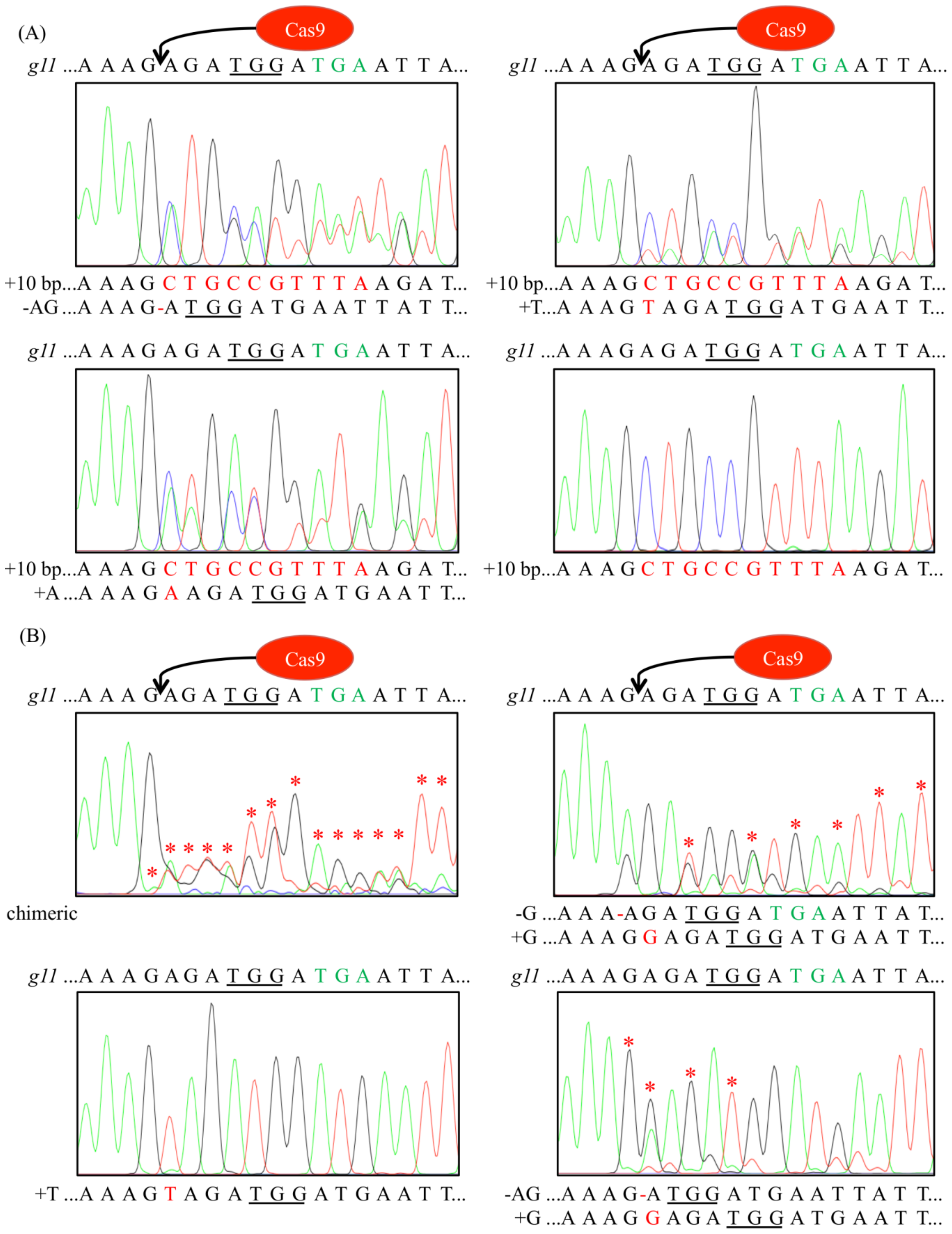
(A)
Cas9
Cas9
gl1 ...A A A G A G A T G G A T G A A T T A...
gl1 ...A A A G A G A T G G A T G A A T T A...
+10 bp...A A A G C T G C C G T T T A A G A T...
-AG ...A A A G-A T G G A T G A A T T A T T...
+10 bp...A A A G C T G C C G T T T A A G A T...
+T...A A A G T A G A T G G A T G A A T T...
gl1 ...A A A G A G A T G G A T G A A T T A...
gl1 ...A A A G A G A T G G A T G A A T T A...
+10 bp...A A A G C T G C C G T T T A A G A T...
+A ...A A A G A A G A T G G A T G A A T T...
+10 bp...A A A G C T G C C G T T T A A G A T...
(B)
Cas9
Cas9
gl1 ...A A A G A G A T G G A T G A A T T A...
gl1 ...A A A G A G A T G G A T G A A T T A...
chimeric
-G ...A A A-A G A T G G A T G A A T T A T...
+G ...A A A G G A G A T G G A T G A A T T...
gl1 ...A A A G A G A T G G A T G A A T T A...
gl1 ...A A A G A G A T G G A T G A A T T A...
+T ...A A A G T A G A T G G A T G A A T T...
-AG ...A A A G-A T G G A T G A A T T A T T...
+G ...A A A G G A G A T G G A T G A A T T...

Supplementary Figure S4: Exemplary sequencing histograms of T4 generation plants with (A) and without (B) trichomes. All plants carrying trichomes contain at least one functional copy of the *GL1* gene with the codon-modified 10 bp integrated in the Cas9 generated DSB. Sequence of the dysfunctional *gl1* gene is given on top of the sequencing histogram for comparison. Multiple peaks that indicate chimerism and unfixed mutations are marked with red asterisks. Peak colors: Adenine = green, cytosine = blue, guanine = black, thymine = red; PAM sequence underlined, premature STOP codon marked in green letters.

1.2 Supplementary Tables

Table S1: Primer Sequences.

Primer	Sequence (5' to 3')
FH190	GTTTGCACGACTAATACACTTATG
FH210	ATTGGAAGAGATGTGGGAAAAGAGA
FH211	AAACTCTCTTTTCCCACATCTCTTC
FH214	AAAGACCATCTGAATTTGGGG
FH215	TAAGCACGTGTCACGAAAACC
FH217	GGGGACAAGTTTGTACAAAAAAGCAGGCTTCACCGTTGACCTGCAGGCACG
FH218	GGGGACCACTTTGTACAAGAAAGCTGGGTGTTTAAACTATGAGGGTCGTACG
FH223	AAAGCTGCCGTTTAAGATGG
FH240	GAGCTTTGGGTACGTCACCCACTTTGTTGTATAAATAACTAAAAATAATATG
FH241	TGACTTGAAGTACACTCATATTAAATTCATACCATATAACATAAATTATAAC
FH265	TCTACTTGCACAATGAAATATATTC
FH266	CATACGGTTCAGGTTGTGGA
FH271	GTGACGTACCCAAAGCTCTG
FH278	GGGGACAAGTTTGTACAAAAAAGCAGGCTTCACCTAGGGCGAATTGGCGGAA
FH279	GGGGACCACTTTGTACAAGAAAGCTGGGTGAGCGGGCAGTGAGCGGAA
FH313	CGGAACTAACTCTGTGGGATG
FH314	CCTCAGCGAGATCGAAGTTAG

Table S2: Sequence analysis of T4 generation plants. Given is the number of progeny plants from each trichome-bearing T3 parental plant with a given phenotype (with or without trichomes) and a given genotype (containing a 10 bp insertion or other genotypes). The genotype ratios do not differ significantly from an expected genotype ratio of 1:2:1 as calculated by chi-squared test.

	**Progenitor T3 plant**		
	**#1**	**#2**	**#3**
**With trichomes** **+10 bp / +10 bp**	13	9	9
**With trichomes** **+10 bp / other**	18	13	20
**With trichomes** **other / other**	0	0	0
**Without trichomes** **+10 bp / +10 bp**	0	0	0
**Without trichomes** **+10 bp / other**	0	0	0
**Without trichomes** **other / other**	8	8	11
**$\chi 2$ value**	1.5128	0.6	0.2
**P value**	> 0.1	> 0.5	> 0.9
